# Bacterial diversity in Icelandic cold spring sources and in relation to the groundwater amphipod *Crangonyx islandicus*

**DOI:** 10.1371/journal.pone.0222527

**Published:** 2019-10-02

**Authors:** Ragnhildur Guðmundsdóttir, Agnes-Katharina Kreiling, Bjarni Kristófer Kristjánsson, Viggó Þór Marteinsson, Snæbjörn Pálsson

**Affiliations:** 1 Faculty of Life and Environmental Sciences, University of Iceland, Reykjavík, Iceland; 2 Department of Aquaculture and Fish Biology, Hólar University, Sauðárkrókur, Iceland; 3 Matis ohf./Icelandic Food and Biotech R&D, Reykjavík, Iceland; 4 Faculty of Food Science and Nutrition, University of Iceland, Reykjavík, Iceland; Free University of Bozen-Bolzano, ITALY

## Abstract

*Crangonyx islandicus* is a groundwater amphipod endemic to Iceland, considered to have survived the Ice Ages in subglacial refugia. Currently the species is found in spring sources in lava fields along the tectonic plate boundary of the country. The discovery of a groundwater species in this inaccessible habitat indicates a hidden ecosystem possibly based on chemoautotrophic microorganisms as primary producers. To explore this spring ecosystem, we assessed its microbial diversity and analysed whether and how the diversity varied between the amphipods and the spring water, and if was dependent on environmental factors and geological settings. Isolated DNA from spring water and from amphipods was analysed using metabarcoding methods, targeting the 16S rRNA gene. Two genera of bacteria, *Halomonas* and *Shewanella* were dominating in the amphipod samples in terms of relative abundance, but not in the groundwater samples where *Flavobacterium*, *Pseudomonas* and *Alkanindiges* among others were dominating. The richness of the bacteria taxa in the microbial community of the groundwater spring sources was shaped by pH level and the beta diversity was shaped by geographic locations.

## Introduction

*Crangonyx islandicus*, Svavarsson and Kristjánsson 2006, is one of two endemic groundwater amphipod species in Iceland [1, 2]. The Icelandic groundwater amphipods are considered to have become geographically isolated when the groundwater in pre-Iceland got disconnected from Greenland during the submergence of the land bridge between the two islands around 15 million years ago [2]. Genetic variation and distinct mitochondrial DNA lineages of the species within Iceland supports this scenario [3]. As Iceland was repeatedly covered by an ice sheet during the Ice ages for the last 2.7 Myrs [4], it is likely that *C*. *islandicus* survived in subglacial refugia, most likely along the tectonic plate boundary [3]. At the same time, the flora and fauna in Iceland are mostly considered to have colonized the island after the last glacial maximum, 10–15 thousand years ago [5]. How the amphipods survived in this sub-glacial refugia is unknown and it is likely that an unexplored ecosystem exists in Icelandic groundwater. Due to their inaccessibility, groundwater ecosystems in Iceland have not been studied thoroughly but some work has been done on their natural access point at the surface in springs e.g. [6]and the groundwater amphipods were first discovered in 1998 [2]. The amphipods have almost exclusively been found in spring sources at the edge of lava fields formed after the last glacial period of Ice age, or during the last 11 000 years e.g. [3].

Microbes play a critical role in every ecosystem where they provide services as primary producers, decomposers and, in general, recycle nutrients, that in turn become available for other organisms. In groundwater systems this is especially true as microbes, mainly found as biofilm, are considered to be a major source of food and energy for meio- and macrofauna [7, 8]. Bacteria and archaea are thus of special interest, being the base of the food web within these systems. Due to inaccessibility these groundwater aquifer systems are commonly studied at their natural access points, i.e. springs and caves. There we can assume that bacteria that are found suspended in the water represent the community within the aquifer, becoming suspended when the biofilm sheds. [9, 10]. Biofilms are formed when microbes attach to each other or surfaces and form a matrix like structure, composed of extracellular products [11]. Over time, a microbial community will grow within this matrix and as new inhabitants colonize it through a species sorting process the biofilm community may differ from the community in the surrounding water [12].

Cold groundwater spring sources constitute a stable environment where chemical and physical properties fluctuate only marginally [13, 14]. The water temperature in most cold springs follow the mean annual air temperature of the region while warmer springs would indicate a geothermal influence [15]. The primary abiotic factor affecting these systems is likely the lack of light, resulting in low biodiversity and productivity [16]. Groundwater ecosystems are most often controlled bottom up, whereas nutrients and carbon flux are considered to penetrate the system from the surface, and are generally thought of as being heterotrophic in nature [17, 18]. Furthermore, food webs of groundwater systems are usually simple, with few trophic links [17]. There has, however, been growing evidence that subterranean ecosystems with more complex food webs can be self-sustainable, independent of light energy, with primary production originating in chemoautotrophic microbial activity. Examples of this are the Movile cave system in Romania [19, 20], the Ayyalon cave in Israel [21, 22] and the Frasassi cave system in Italy [23, 24]. The water in these cave systems is rich in H_2_S and the bedrock is made of limestone, CaCO_3_ [21, 25, 26] which differs greatly from the groundwater systems in Iceland, which consist predominantly of basalt. Basalt has the potential of being an energy source for microbial growth as it is rich in minerals such as iron (Fe), sulphur (S) and manganese (Mn) providing opportunity for organic growth from chemolithoautotrophic bacteria [27–30]. Given the geological settings of the bedrock type where the amphipods are found, bacteria capable of utilizing sulphur and iron are of special interest.

Besides being a natural access point to groundwater aquifer systems, springs constitute an interesting habitat as an ecotone between groundwater, surface water and the terrestrial ecosystem [31]. Spring sources are biodiversity hotspots, especially for invertebrates, bryophytes, diatoms and cyanobacteria [32, 33] and they form a discrete habitats in the landscape and are therefore good for testing spatial patterns of community composition e.g. [34].

Iceland is rich in groundwater due to high precipitation rates [35, 36] and large glaciers [37]. As the basaltic lava fields within the volcanic active zone are highly porous [38] the groundwater can be influenced by surface waters [37]. These surface water influences are likely to vary according to the aquatic habitat created at the spring source. In some cases, the springs form a pool at the source (limnocrene) while in others a stream is formed (rheocrene). The cold-water springs located within the volcanic active zone in Iceland are categorized as fennoscandic mineral rich springs according to the EUNIS classification (C2.111 European Environment Agency) and have a high conservation value. In Iceland, springs have been studied to some extent regarding their invertebrate community structures [6, 39, 40] but not for microbial communities in cold groundwater.

Using environmental DNA (eDNA) and metabarcoding [41] we focused here on two main objectives:1) Assessment of microbial diversity in cold groundwater spring sources in the volcanic zone of Iceland, both suspended in the water current and in a biofilm. Furthermore, we examined which factors shape the microbial communities in this habitat. This part of our study enhances our understanding of the microbial community in cold springs. 2) Whether distinct groups of chemolithoautotrophs occur within the microbial community of the springs, and whether specific bacteria are associated to the groundwater amphipods. Finding those could potentially explain how the amphipods survive in the groundwater ecosystem, even in subglacial refugia during the glacial periods of Ice Age in Iceland.

## Materials and methods

### Sampling and sample preparation

#### Water samples

**Fig 1:**
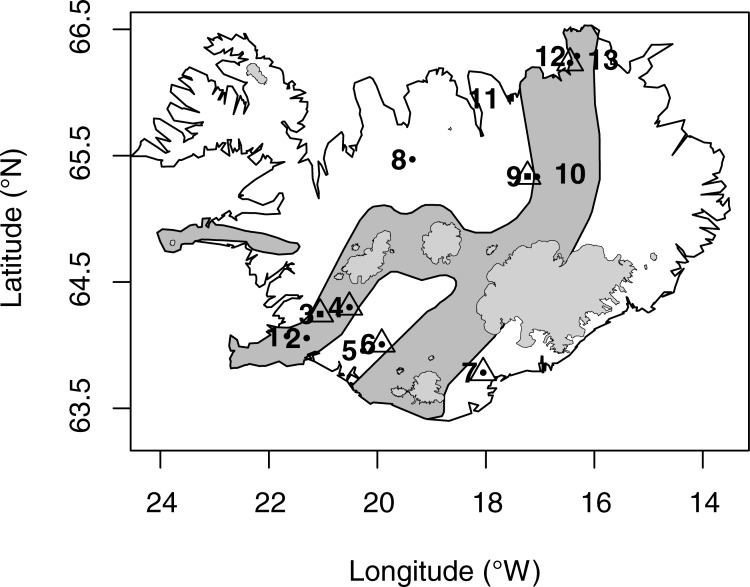
Map of the sampling sites within Iceland. Squares represent locations where amphipods were sampled, triangles locations with glass bead samples, water samples were collected at all sampling locations. The volcanic active zone is represented in dark grey.

In the summers of 2014–2015, 13 water samples were collected from the spring source of thirteen cold groundwater springs in Iceland (Fig 1). The springs were characterized by stable environmental conditions and different sites can thus been regarded as biological replicates e.g. for spring type, more samples per site over all would have provided a better estimate of the variation due to sampling. The variation in environmental variables was measured and considered in the statistical analysis (see below). Eleven of the springs were cold springs (temperature ranging from 2.5–5.3°C), located in lava fields throughout the volcanic zone. Two warmer springs were included to get an indication of the effect of higher temperature, one within the volcanic active zone at the geothermal area Hengill SW Iceland (11.5°C) and one outside the zone at Steinsstaðir, North Iceland (42.6°C, Fig 1, Table 1). The springs were of two different types, eight rheocrene (stream-forming) and five limnocrene (pool-forming). Seven samples were collected in the active current at the spring source, and six samples were taken about two meters downstream of the spring source (referred to as surface onwards, Table 1). Each sample consisted of five litres of water, which were either pumped with a drill pump, through a hose, into bottles or the bottles were lowered into the water. The bottles had been washed with HCl (10%) and autoclaved. The hose was flushed with water from the sampling location for 5 minutes prior to sampling. The samples were stored in a cooler for up to 24 hours, when they were filtered through Sterivex^TM^ filter (GV Durapore, pore size 0.22 μm cellulose membrane filter, Millipore Corporation, Billerica, USA). The filters were stored in dark and frozen at -25°C until further analysis.

**Table 1:** Sample information with locations from a study on biodiversity of bacteria in Icelandic springs.

Sample name	Reads	Lat N	Lon W	Area	Alt	Temp (°C)	pH	Spring type	Source/ Surface	Fish
1.W.15	1847	64.071	21.669	SW	121	5.3	8.7	R	O	0
2.W.15	17231	64.057	21.304	SW	406	11.5	7.4	R	O	0
3.W.14	2846	64.245	21.055	SW	109	3.0	9.0	L	S	1
3.G2.14	3727	64.245	21.055	SW	109	3.0	9.0	L	S	1
3.W.15	23428	64.245	21.055	SW	109	3.6	8.7	L	O	1
3.A1.12	2852	64.245	21.055	SW	109	NA	NA	L	S	1
3.A2.12	3226	64.245	21.055	SW	109	NA	NA	L	S	1
4.W.14	6613	64.289	20.512	S	184	2.5	9.3	L	S	0
4.G1.14	31024	64.289	20.512	S	184	2.5	9.3	L	S	0
4.G2.14	19464	64.289	20.512	S	184	2.5	9.3	L	S	0
5.W.15	33015	63.957	20.265	S	78	4.7	7.9	R	O	1
6.W.14	69997	64.008	19.919	S	130	5.0	7.9	R	S	1
6.G1.14	20558	64.008	19.919	S	130	5.0	7.9	R	S	1
6.G2.14	17213	64.008	19.919	S	130	5.0	7.9	R	S	1
7.W.14	2974	63.782	18.05	SE	30	5.5	NA	R	S	0
7.G1.14	10545	63.782	18.05	SE	30	5.5	NA	R	S	0
7.G2.14	37288	63.782	18.05	SE	30	5.5	NA	R	S	0
8.W.15	21694	65.469	19.357	NW	62	42.6	8.5	R	S	0
9.W.14	18295	65.336	17.232	N	382	3.7	9.0	L	S	0
9.G1.14	5885	65.336	17.232	N	382	3.7	9.0	L	S	0
9.G2.14	20594	65.336	17.232	N	382	3.7	9.0	L	S	0
9.A1.12	20323	65.336	17.232	N	382	NA	NA	L	S	0
9.A2.12	7745	65.336	17.232	N	382	NA	NA	L	S	0
10.W.15	23419	65.334	17.057	N	441	5.5	9.0	R	O	0
11.W.15	47749	65.954	17.545	N	8	3.8	7.4	L	O	1
11.A1.13	58143	65.954	17.545	N	8	NA	NA	L	S	1
11.A2.13	38143	65.954	17.545	N	8	NA	NA	L	S	1
12.W.15	7235	66.258	16.401	NE	28	4.1	8.0	R	S	1
13.G1.14	3412	66.274	16.408	NE	9	4.0	7.6	L	S	1
13.G2.14	23884	66.274	16.408	NE	9	4.0	7.6	L	S	1

Sample name is composed of a code for map location (1–13) as in Fig 1, sample type (A, W, G) and year (2012–2015). Map location code: 1: Lækjabotnar Reykjavík, 2: Hengill, 3: Þingvallavatn, 4: Miðhúsaskógur, 5: Lækjabotnar, Holtum, 6: Galtalækur, 7: Kirkjubæjarklaustur; 8: Steinsstaðir, 9: Svartárvatn, 10: Hagalækur, 11: Sandur, 12: Presthólar, 13: Klapparós. Sample types are W: Water, G1: Glass beads (treatment 1), G2: Glass beads (treatment 2), A1 and A2: Amphipod specimens 1 and 2 respectively. Reads stands for number of paired-end reads. Lat and Lon are latitude and longitude. Area: Geographic regions within Iceland, south west (SW), south (S), south east (SE), north west (NW), north (N) and north east (NE). Alt: Altitude (m above sea level). Spring types are R: Rheocrene and L: Limnocrene. Source (S) and Surface (O) refer to sampling location within springs. Fish: absence (0) and presence (1). Rarefication level was 1847.

#### Glass bead samples

Biofilm was collected at the seven spring sources sampled in 2014 (Table 1), by an in-situ incubation glass beads (4 ± 0.3 mm in diameter, Carl Roth GmbH, Karlsruhe, Germany). The glass beads serve as inert support for biofilm formation and could collect microbes similar to the biofilm the amphipods are feeding on. The beads were placed in net envelopes with mesh size 1 mm, 120 beads in each, with a surface area of 60.3 cm^2^ and weight of 10 ± 0.1 g. The net envelopes were placed as far deep into the spring source as possible, ranging from 2 to 10 cm, in the middle of the groundwater current. As the roughness of the surface of the glass beads can facilitate the growth or attachment of microbes e.g. [42], the glass beads were treated with one of the following two methods: Treatment 1): depolishing using a base, Armour Etch® (sodium difluoride) for 10 minutes and treatment 2) depolishing with sandblasting for 20 minutes. Each treatment type was put in a separate net envelope. Before the glass beads were put out in the field the net envelopes were rinsed twice with distilled water and autoclaved for 20 minutes at 132°C, according to manufacturer instructions. At each sampling site net envelopes, with both types of the depolished glass beads, were placed and incubated in the field for eight to ten weeks. When retrieved, the net envelopes were put into 30 ml of sterile water and shaken for 20 minutes, 5 ml of the water was put into tubes for cell count. The rest was filtered through Sterivex^TM^ filters (0.22 μm). Both tubes and filters were frozen at -25°C until further analyses.

#### Amphipod samples

A sample of six specimens of *Crangonyx islandicus* were included in the analysis. The amphipods had been caught at three of the springs for another study but were included here for comparison with the bacterial community in the springs (Table 1). These three springs were at Sandur and Lake Svartárvatn both in North Iceland and one at Lake Þingvallavatn, South West Iceland (Fig 1 and Table 1). The amphipods selected fell within two of the five evolutionary distinct mtDNA linages within the species from these two areas [3]. The amphipods were sampled with electro-fishing gear in the spring source, where amphipods within the electrical range get shocked and float out from the spring with the current and were then collected with a dip-net and stored in 96% ethanol right after sampling. The specimens from Lake Þingvallavatn and Lake Svartárvatn were collected in summer 2012 and the specimens from Sandur in summer 2013.

#### Environmental variables

At each sampling site, temperature was measured and in 2015 pH, oxygen saturation and water conductivity were also measured. Due to stability of the spring source environment e.g. [43] we infer the environmental variables to the 2014 samples as well. The elevation above sea level in meters, presence of fish and geographical area was recorded (Table 1). Geographical areas were classified based on divergent mtDNA lineages within *C*. *islandicus* [3], three in South Iceland from west to east (SW, S, SE), and two in North Iceland (N, NE). Based on the distinct mtDNA lineages the areas are considered to present different groundwater basins. One sample was in a geographical area outside the distributional range of *C*. *islandicus* (8.W.15), in Skagafjörður, Northwest Iceland (NW).

To assess variation in chemical composition of the springs, 200 ml of water were collected from seven springs sampled in 2014, stored in polypropylene bottles prewashed with HCl (10%) and kept frozen till further analysis. Chemical elements (S1 Table) in the water samples were measured with ICP-MS at Matís, Food and biotech R&D, Iceland and nutrients (NO_2_, NO_3_ and PO_4_) at the Marine Research Institute in Iceland as described in Bartrones et. al. [44].

Permits for fieldwork were obtained from landowners and the director of the national park of Þingvellir.

### Unicellular biomass

To assess the unicellular biomass at the springs, 5 ml of water from the source or from beads of seven springs (Table 1) were fixed with glutaralderhyde (Sigma-Aldrich, St. Louis, MO, USA), 0.1% final concentration, and later analysed with flow cytometry. Right after fixation the 5 ml tubes were stored in the dark on dry ice and brought to the lab and frozen for storage at -25°C. The samples (n = 20) were thawed at room temperature and stained in 1:1000 solution of Sybrgreen I (Molecular Probes, Eugene, OR, USA). Samples were incubated in the dark for 20 minutes and run through BD FACSAria^TM^(BD Biosciences, Franklin Lakes, NJ, USA) flow cytometer at calibrated flow rate for 2 minutes. Forward- and right-angle light scattering, and DNA fluorescence data were collected and analysed according to the protocol provided by the producers of the CountBright^™^ absolute counting beads (Invitrogen, Carlsbad, CA, USA. Manual: https://www.thermofisher.com/order/catalog/product/C36950). Cell counts for different glass bead treatments were compared using the nonparametric Wilcoxon Ranked Sum test due to uneven variance of the samples. Correlation for cell count and read number was tested with linear regression.

### DNA extraction

Total DNA from the water samples and the glass beads was extracted from the Sterivex filters as described in Neufeld *et al*. [45], where the filters were washed in a SET buffer and lysozyme solution, and the samples digested with proteinase K (all reagents from Sigma-Aldrich, St. Louis, MO, USA). The DNA was then rinsed by standard phenol-chloroform extraction (all reagents from Sigma-Aldrich, St. Louis, MO, USA) using a rotating hybridization oven (Hybridiser HB-1D, Techne, Bibby Scientific Limited, Staffordshire, UK). The DNA from the amphipods was extracted directly from whole animals in 6% Chelex 100, (Bio-Rad Laboratories Inc, Hercules, CA, USA) including proteinase K [3].

### Amplification and sequencing

Bacteria species diversity was assessed by amplifying a 464 bp (base pair) fragment from the V3 to V4 region of the 16S rRNA gene using the primer pair: S-D-Bact-0341-b-S-17 and S-D-Bact-0785-a-A-21 [46, 47]. A 25 μl PCR mix contained 0.2 μM dNTPs, 1X Phusion^®^ HF buffer containing 1.5 mM MgCl_2_, 3% DMSO, 0.32 μM primers, 0.01 u μl^-1^ Phusion^®^ High-Fidelity DNA polymerase (New England BioLabs Inc., Ipswich, MA, USA) and 0.3 ng μl^-1^ of template. Additional MgCl_2_ was added to the solution to make the final concentration 2.7 mM. The PCR conditions were 98°C 30 sec, followed by 30 cycles of 98°C 10 sec, 52°C 30 sec, 72°C 30 sec, with a final elongation step at 72°C for 7 min.

Archaea were amplified with a similar protocol, but without DMSO and with an annealing temperature of 50°C. The primer pair was S-D-Arch-0519-a-S-15 and S-D-Arch-1041-a-A-18 [47], amplifying a 540 bp fragment from the V4 to V6 region of the 16S rRNA gene.

Amplifications were performed in DNA Engine Tetrad2 Peltier Thermal Cycler (Bio-Rad Laboratories Inc, Hercules, CA, USA) and the PCR products were inspected by agarose gel electrophoresis. Both positive and negative control was conducted for each PCR reaction.

The PCR products were purified and concentrated using illustra^TM^ GFX^TM^ PCR DNA and Gel band Purification Kit (GE Healthcare, Chicago, IL, USA), followed by an indexing procedure using Nextera XT Index Kit (Illumina, Inc., San Diego, CA, USA), all according to the manufacturer’s instructions. The size of the DNA fragments was estimated using the Agilent High Sensitivity DNA kit (Agilent Technologies, Inc., Santa Clara, CA, USA).

Library quantification, normalization, pooling and library denaturing followed the 16S rRNA gene Metagenomic Sequencing Library preparation protocol (Part # 15044223 Rev. B). DNA amplicon sequencing was performed on a MiSeq Illumina platform.

### Taxonomic assignment of DNA sequences

The raw sequencing data from the Illumina MiSeq was analysed using OBITools [48]. The paired end reads were merged using a criterion of a minimum of 40 bp overlap. To reduce rare variants, sequences that were represented with ten or more copies and at the minimum of 80 bp length, were kept for taxonomic assignation and further analyses. To reduce computational time, sequences were clustered into unique sequences clusters (100% identical) and a representative sequence of each cluster was selected for taxonomic assignment using the 128 release of the SILVAngs database [49, 50]. As the hit can be divergent from the query sequence (e.g. >5% divergence) or that the reported identity is low due to partial BLAST alignments, the hits were accepted if average of sequence identity and alignment coverage was larger than 93, a threshold which has been found empirically to give reliable results (SILVAngs user guide: https://www.arb-silva.de/ngs/service/file/?file=SILVAngs_User_Guide_15_12_15.pdf). An in-house script was used for preparing the output from SILVAngs for analysis in R, taking into account the frequency observed for each cluster. The nucleotide sequence data reported are available in the European Nucleotide Archive under the accession number PRJEB32879. Other data can be accessed at https://doi.org/10.6084/m9.figshare.9773366.v1 or found in S1–S4 Tables.

### Statistical analysis

For diversity assessment, the samples were rarefied using the rrarefy function in the vegan package [51] in R [52] to 1847 reads per sample, in order to retain as many sampling sites as possible. Numbers close to 2000 reads per sample have been demonstrated as sufficient to detect community patterns among samples [53].

The frequency of the major phyla was summarized with a barplot and a Venn diagram, were used to summarize the number of co-occurring and unique OTUs linked to the amphipods and in the water samples and the glass beads. The plots were produced in R using the VennDiagram package [54].

Chemical composition among sampling sites were tested for difference using *permanova* via adonis in the vegan package in R [51]. Alpha diversity was estimated using the Shannon index, Pielou’s evenness and species richness as they may reflect different aspects of the data, e.g. Pielou´s evenness scales the Shannon diversity index with the number of taxonomic units e.g. [55]. Independence of the indices, or whether they differ for our data was evaluated with pairwise correlations. A multiple linear regression was conducted to reveal how much of the variance in the alpha diversity could be explained by the environmental variables (Table 1). A stepwise model selection was conducted with the step command implemented in R. Model diagnostics plots were used to check whether the assumptions were met for the models, e.g. by exploring the distributions of the residuals and variance inflation factor was calculated to evaluate collinearity among the explanatory variables. Variation in cell counts were analysed in respect to the read numbers (log-transformed) and the diversity estimates by linear regression.

Differences between samples, the beta diversity, was summarized with Bray Curtis dissimilarity and visualised with non-metric multidimensional scaling (NMDS) and Principal Coordinate Analysis (PCoA) using the function metaMDSin the vegan package[51] and pcoa in the ape package respectively [56]. As the NMDS uses ranked orders of samples excluding the abundances we consider that to be more conservative than the PCoA and therefore only demonstrate the NDMS figures. To identify the source of variation in taxonomic composition between the samples, permanova was conducted, with 1000 permutations, using the adonis function in the vegan package in R [51]. Firstly, to test the effect of the glass bead treatments. Secondly, to test whether the water samples differed with respect to environmental variables, origin of sample (source and surface) and geographic location (see Table 1). Lastly the microbiome of the amphipods was compared to the composition in the water and bead samples from the same location, and between bacteria composition on the amphipods and the water and bead samples from all locations. The association of chemical data with the beta diversity summarized with the NMDS ordination was analysed with the envfit function in vegan [51].

## Results

### Cell counting

In total 20 samples were processed for cell counting, twelve glass bead samples (six of each treatment type) and eight water samples. No difference was observed for number of cells between the bead types (Wilcoxon W = 21, p = 0.70). The average number of cells for glass beads was nominally higher than in the water samples (S2 Table) although not statistically different (Wilcoxon W = 73, p = 0.057).

Cell number was a moderate predictor for total reads (r^2^ = 0.27, b = 0.25, p = 0.04). However, taxa richness or total number of OTUs per sample showed no relationships with cell numbers, also after taking the sample types into account (p > 0.05).

### Sequencing results

From the amplification of bacteria, 685,399 sequences were obtained. Almost all were of bacteria origin (685,399 or 99.7%), representing 226 assigned taxa in 30 samples. The other sequences (1,863 in total) obtained with the bacteria primer pair were of archaea origin. 74,165 sequences were of chloroplast origin and as they present symbiotic organelles with eukaryotes they were omitted from further analyses as well as mitochondria sequences that were six in total.

In total 174,708 assigned sequences were obtained with the archaea primers, but only 1,443 (0.08%) were of archaea origin whereas 171,516 (98.2%) were from bacteria and 1,566 (0.09%) from eukaryotes. These 1,443 archaea sequences represented ten taxa in seven samples from five locations. As the results obtained for the archaea were poor in quantity, we report only the main groups observed but do not analyse further the variation in the archaea samples. Two of the taxa were found in the glass bead samples but not from the amphipod samples. The archaea OTUs with most reads were Woesarchaeota (DHVEG-6), Thaumarchaeota Marine Group I and South Gold Mine Group1 (SAGMG-1).

### Diversity in springs

**Fig 2:**
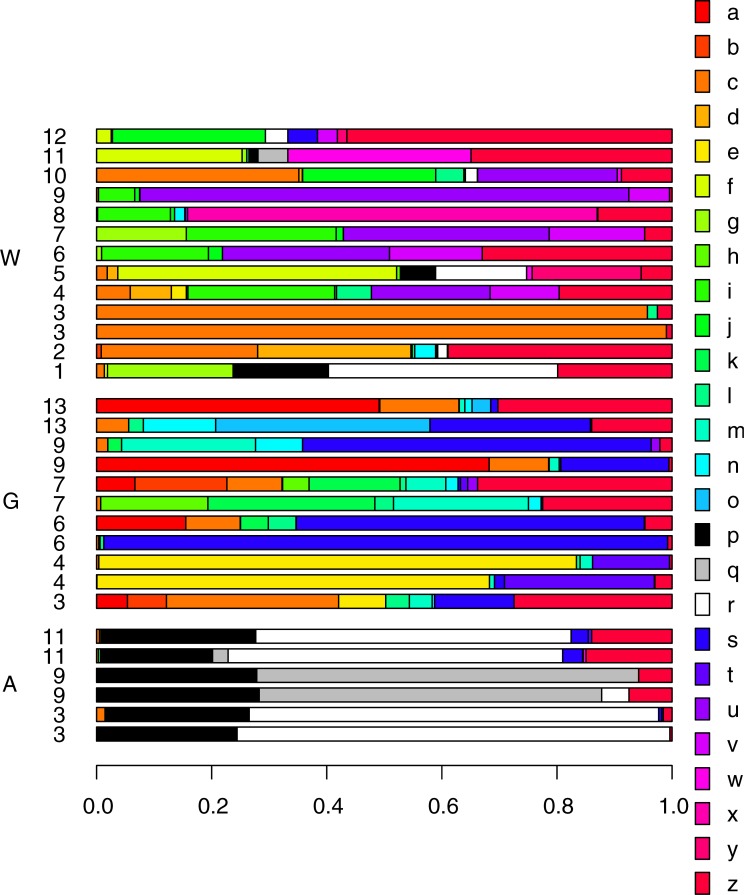
Bacteria taxa with relative abundance more than 20% in all sample types. A = Amphipod samples, G = Glass bead samples, W = Water samples. Numbers refer to sampling locations (same as in Fig 1). Legend: a = Cytophaga, b = Cytophagaceae uncultured, c = Flavobacterium, d = Chamaesiphon, e = Tychonema, f = Bacillus, g = Omnitrophica, h = Rhizorhapis, i = Sphingobium, j = Sphingomonas, k = Aquabacterium, l = Polaromonas, m = Rhodoferax, n = Comamonadaceae uncultured, o = Sandaracinaceae uncultured, p = Shewanella, q = Halomonadaceae, r = Halomonas, s = Alkanindiges, t = Perlucidibaca, u = Pseudomonas, v = Stenotrophomonas, w = Luteolibacter, x = Limnothrix, y = Citrobacter, z = Taxa <20%.

In total, 158 taxa of bacteria were observed in the spring community for both water samples and in the glass bead samples. The dominating phylum (Fig 2) in terms of relative abundance was Proteobacteria (11–99%), except for Lake Þingvallavatn where Bacteroidetes was dominating, mainly represented by the genera Flavobacterium with 97–100%. No difference in community composition was observed between the two glass bead treatments incubated in the spring sources (p = 0.36) and they were thus treated as one in further analysis.

The alpha diversity of bacteria in the springs (Table 2), represented by the Shannon index, showed indication of a negative association with pH level (b = -0.5, p = 0.059). The taxa richness (Table 3) decreased with increased pH level and with the presence of fish (b = -5.6, p = 0.02). Other variables tested did not influence the alpha diversity (p > 0.4). Although the Pielou´s evenness index was highly correlated with the Shannon index, its variation was independent of the environmental variables measured (p > 0.6).

**Table 2:** Alpha diversity measurements for all sample types.

	Shannon	Pielou’s	Richness
Amphipods	1.1±0.4	0.4±0.1	19.5±13.7
Glass beads	1.4±0.7	0.5±0.2	16.5±12.9
Water	1.5±0.8	0.5±0.2	22.2±15.9

Shannon Wiener diversity index, Pielou’s evenness and richness as number of taxa observed in each sample type.

**Table 3:** A linear regression of taxa richness.

		Estimate	Std. Error	t value	p
Richness	Intercept	14.7	4.0	3.7	0.0018
	Fish	-0.5	0.3	-1.8	0.09
	(log)pH	-5.6	1.8	-3.1	0.007

A linear regression of taxa richness as a function of pH and presence of fish (r^2^ = 0.35, df = 17).

The beta diversity among springs varied in relation to different geographical areas, temperature, source and surface, presence of fish and in relation to different sample types (Table 4A). The cyanobacteria *Limnothrix* increases as the temperature increases while they cyanobacteria *Tychonema* decreases along with the photosynthetic β-Proteobacteria *Rhodoferax*. In the springs where fish were present *Flavobacterium* and *Alkanindiges* are common taxa while in springs which don´t harbour fish *Bacillus* and *Halomonas* were more common. Permutational multivariate analysis of variance showed that the greatest difference (25%) was related to the different geographical areas represented by different watersheds (*permanova*, p = 0.001) although the chemical composition among tested sites did not differ (*permanova*, p = 0.39). The second largest difference was related to sample type (14%), as the glass beads had different community composition compared to the water samples (p = 0.001), for details see Table 4. Of the 2% most common taxa for both water samples and glass bead samples nine out of 21 were shared between the two types, the most frequent one being *Flavobacterium* (7.5% relative abundance on glass beads but 20.5% in water samples). For overview of all taxa amplified see S3 Table.

**Table 4:** Source of variation for beta diversity.

Source of variation	df	SS	MS	F	R^2^	p
a)						
Sample type	1	1.4	1.4	5.1	0.14	0.001
Source/surface	1	0.7	0.7	2.6	0.07	0.002
Spring type	1	0.4	0.4	1.5	0.04	0.098
Temp	1	0.5	0.5	1.8	0.05	0.002
Elevation (m)	1	0.3	0.3	1.2	0.03	0.225
Fish	1	0.7	0.7	2.5	0.07	0.004
Area	5	2.4	0.5	1.8	0.25	0.001
Residuals	12	3.3	0.3		0.34	
Total	23	9.8			1.00	
b)						
Sample type	2	3.4	1.7	5.0	0.27	0.001
Residuals	27	9.1	0.3		0.73	
Total	29	12.4			1.00	
c)						
Sample type	2	2.6	1.1	4.7	0.47	0.001
Residuals	10	2.6	0.3		0.53	
Total	12	4.8			1.00	

Difference in composition of the different taxonomic units (beta-diversity) among samples from different springs and sample units. The ratio of variance among sites and within sites was tested with permanova using the adonis function in the R-package vegan [51]. a) Bacterial community in springs (water and glass bead samples) tested with environmental factors, b) bacterial communities in all sample types among all sampling sites and c) for sites where all sample types were collected. Number of permutations: 999. A: amphipods, W: water and G: glass beads.

Removing the warm spring sources from the dataset did not alter the signal for source of variation for the beta diversity (S4 Table).

### Amphipod microbiome

**Fig 3:**
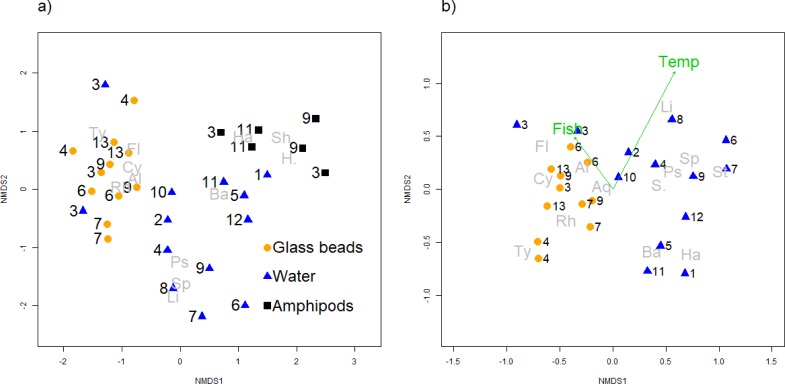
Non-metric multidimensional scaling (NMDS) plot for beta diversity patterns of bacteria in Icelandic springs. Numbers refer to sampling sites (see Fig 1). Abbreviations in grey refer to names of taxa: Al:Alkanindiges, Aq:Aquabacterium, Ba:Bacillus, Cy:Cytophaga, Fl:Flavobacterium, H.:Halomonadaceae, Ha:Halomonas, Li:Limnothrix, Ps:Pseudomonas, Rh:Rhodoferax, Sh:Shewanella, Sp:Sphingobium, S.:Sphingomonas, St:Stenotrophomonas, Ty:Tychonema. A) All sample types (amphipods, water and glass beads, stress = 0.18. B) Amphipods excluded from analysis, stress = 0.20. Temp = temperature, p = 0.002 and fish = presences of fish at the sampling site, p = 0.004.

When all sample types were analysed together for the beta diversity the bacteria community in relation to amphipod differed greatly from the water and the bead samples (Fig 3, Table 4B), indicating that the amphipods harbour a specific microbiome, which is only marginally detected in the water samples (see also Fig 2). When comparing the three sites where all sample types were analysed the difference among samples remained significant (Table 4C).

**Fig 4:**
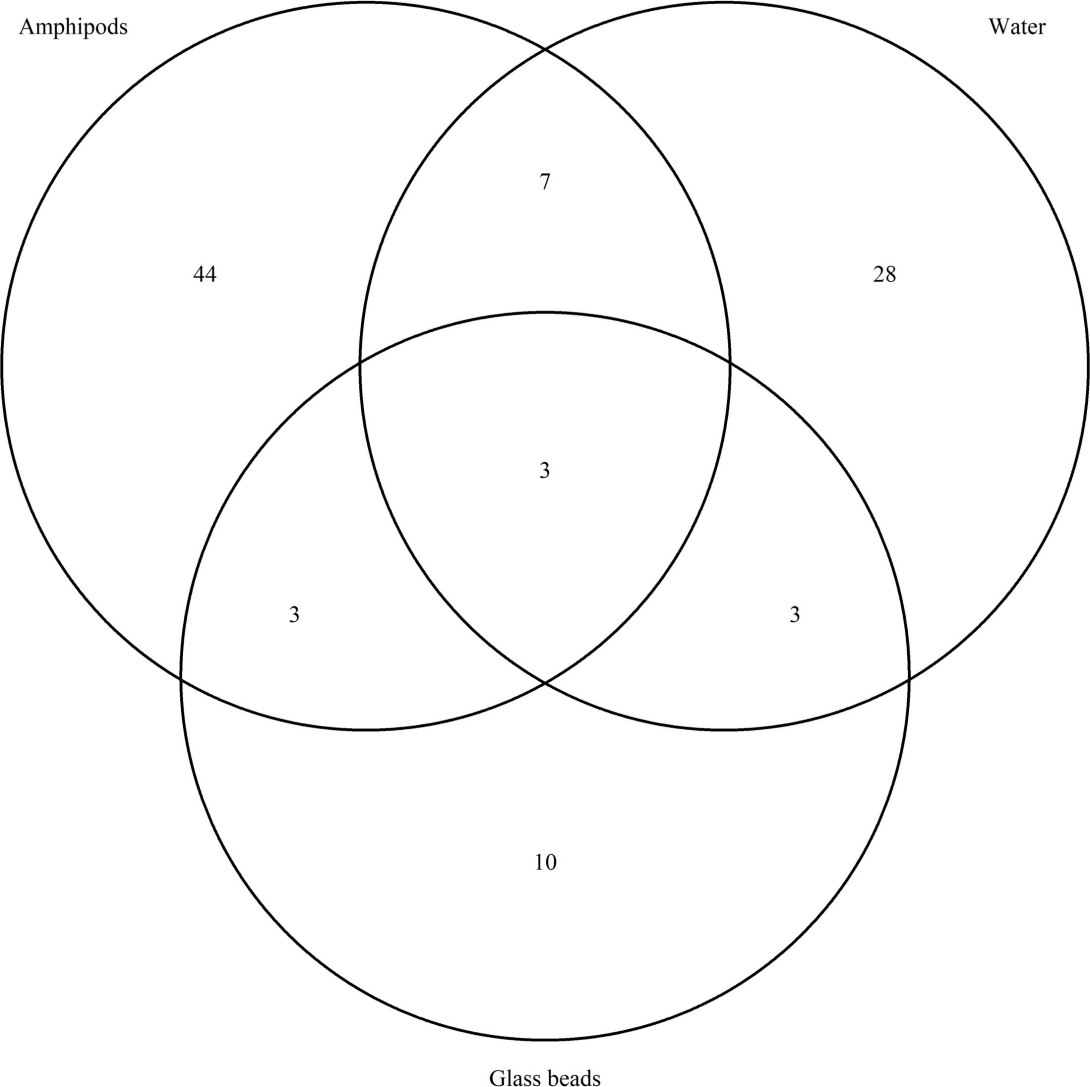
Overlap in taxa between all sample types. Venn diagram showing overlap in taxa from amphipods, water samples and glass beads in collected in Icelandic springs where all sample types were obtained.

Out of 57 bacteria OTUs associated with the amphipods, 44 were unique to the amphipods (Fig 4), but only three were found to be more frequent than 3%. The most frequent ones (65.8%) are bacteria of the family Halomonadaceae. Of those the genus *Halomonas* (44.3%) was found in five of the amphipods, that came from distinct geographic locations. The other OTUs within the Halomonadaceae, (21.4%) did not match to any taxon at lower level than family in the reference database. This variant was found in three amphipods out of six, all from North Iceland. Another abundant taxon (24.9%) found in the amphipods was the genus *Shewanella*. Two distinct types, A and B of *Shewanella* (Table 5) were found in all the amphipods. Considering the sequence divergence between the two OTUs, they may present different species. *Propionibacterium* constituted 1.3% and the remaining bacteria (7.6%) were consisted of 56 OTUs in frequencies less than 1%. For relative abundance of all taxa see S3 Table.

**Table 5:** Genetic diversity of the 16S rRNA within the bacteria genus *Shewanella* sp.

Clade	Nucleotide diversity (π)	Number of sequence variants	Number of reads
A	0.006	5	134
B	0.006	6	295
All	0.022	11	429

## Discussion

The microbial diversity and communities in the spring sources in Iceland vary with respect to geographical areas, presences of fish in the spring and environmental factors such as temperature and pH. The bacteria associated with the groundwater amphipods reveal groups which differ from the environmental samples, indicating a special microbiome associated with the amphipods independent of geographical location. This might reflect any form of symbiotic relationship. Furthermore, we identified few taxa capable of chemolithoautotrophic growth, as described further below, although not in such a great abundance that it would explain the existence of a subterranean ecosystem in Iceland throughout repeated the glacial periods.

### Spring communities

The number of cells in the groundwater springs was a moderate predictor of the read number generated from each sampling location. The counts were independent of the sampling technique, however the glass beads had nominally higher number than the water sample suggesting the build-up of a biofilm on the solid substrate. The overall counts fall within the previous range of cells in groundwater as reviewed by Schmid and Hahn [57] and are less than have been observed in surface water in Lake Þingvallavatn, 3 * 10^5^−10^6^ cells ml^-1^ [58].

Alpha diversity decreased with increased pH in the springs. Other environmental variables such as temperature, conductivity and oxygen concentration did not explain the diversity within the bacterial community at local scale. This may reflect the stable environmental settings of the cold groundwater springs assessed in this study. Similar patterns have been observed previously for microbial communities across various sample types, e.g. in geothermal springs [34] and soil [59] where concentration of protons can change the chemical properties such as the nutrient availability in the environment. The pH of cold springs in Iceland is generally high and more variable than the temperature, ranging from 7–9.3 which can be explained by the basaltic lava rocks, the most common lava type in Iceland. The lowest pH was found in, the warm spring at Hengill in Southwest Iceland and at cold spring in Sandur, Northeast Iceland where the value was 7.4 in both cases. pH lower than neutrality is known to reduce bacteria diversity in soil [60] and in ground-water karst system in central China [61].

Several cold spring bacteria have been identified in springs with special physical and chemical characteristics. These are the iron bacteria *Crenothrix*, *Leptothrix* and *Gallionella* from iron rich springs [32, 62]. We found *Leptothrix* in our spring community samples at two sites (Sandur, North Iceland, and in Kirkjubæjarklaustur, South Iceland). *Crenothrix* and *Gallionella*, were not found although the latter has been found in iron rich springs, harbouring iron oxidizing mats, in Iceland [63]. The presence of taxa such as *Leptothrix* is of interest as it is possibly chemolitotrophic and may have been a key for the survival of the amphipod species. However, their relative abundance, in the sequences was not high. It is thus likely that there may be other species that participate in primary production in these groundwater systems. Groups of cyanobacteria, such as the genera *Chamaesiphon* spp., *Homoeothrix* spp. and *Phormidium* spp. have also been identified as spring taxa [32]. Only *Chamaesiphon* was found in our samples and in low abundance (1% across all samples).

Two genera of carbon fixing bacteria, *Leptospirillum* and *Nitrospira* of the phylum Nitrospira were found in the environmental samples, these are likely candidates in contributing to chemoautotrophic action, as described for the basaltic ocean crust via the Calvin cycle [30]. These genera have been found previously in a study on CO_2_ injection into basaltic lava fields conducted in Iceland [64].

Beta diversity in groundwater spring sources revealed that the community were more alike within geographical areas than among them and this account for the largest difference among the bacterial communities. As the spring sources are stable in terms of physical properties and relatively similar across geographical areas, with two exceptions, we speculate that it could be expected that communities would be similar across areas. This may indicate that factors such as dispersal limitation by distance and even local diversification (through species sorting and stochastic factors) might be shaping the community structure. Dispersal limitation has been detected in geothermal springs in New Zealand where dissimilarity between springs was found to increase with increased geographical distance [34].

Temperature, presence of fish and sampling site within spring (taken at source of the springs or in the surface) also contributed to the difference in the community structure among springs. The influence of temperature was mainly seen in the cyanobacteria *Limnothrix*. Fish might influence the community composition through microbes shed by the fish. The difference between samples at the source and in the surface, might reflect the difference between the groundwater communities and the surface spring community that is likely to be influenced by the open water. It is interesting that the communities change in as short distance as 2 m downstream, which could be related to differences in community compositions of invertebrates between these two areas (Kreiling et al. in prep) and likely increased nutrient load, as primary production through photosynthesis increasingly affects communities downstream. These results, however, need to be taken with a grain of salt, as we did not collect these downstream samples in all springs.

Only few taxa were shared by the glass beads and the water sampled at the source of the springs. The glass bead assembly may reflect admixture of species flowing with the groundwater current into the spring and from the spring water at the surface, whereas the species caught in the water current originate from the ecotone within the dark side of the spring. The bead assembly is affected by a sorting procedure as well, following the colonization process on the solid substrate [42]. Thus, the glass bead community can be considered to be complementary to the community observed in the spring water. However, several bacteria taxa were shared by the amphipods and the glass beads and they could therefore present part of the biofilm which the amphipods may feed on. To which extent the biofilm on the beads represent biofilm within the aquifer, or outside the springs needs to be tested further e.g. by placing the beads further into the spring source, or with other methods used to dislodge the biofilm. This is though a hard task as the spring sources are often narrow or hard to enter for more than just few centimetres.

Only three taxa out of 98, *Aquabacterium*, *Flavobacterium* and *Pseudomonas*, were shared between all sample types. Chloroplast sequences were also found in all sample types, but they were removed from the data set prior to the community analysis. The chloroplasts are of unidentified origin but could come from diatoms which have been found commonly in subterranean habitats [65] and in spring communities e.g. [66]. Finding chloroplast in the groundwater samples might also indicate influences of sunlight and surface impacts, reaching into the groundwater system which is sampled in this study which can be seen as a three-way ecotone between groundwater, surface water and a terrestrial ecosystem [31]. The other taxa detected in this study, either as microorganisms or extracellular DNA may also be of surface origin [31], as they or their DNA could penetrate from the surface with precipitation or from the surface water into the groundwater system through the porous lava.

The amplification of the archaea failed mostly and only few taxa were retrieved from the samples. The most frequent OTUs of the archaea was *Woesearchaeota*, which has been found to dominate archaeal communities in high-altitude oligotrophic lakes [67]. Further studies are needed to explore the archaea species, e.g. with other primer pair [68], nested PCR [69] or whole genome sequencing [70]. It is of interest to explore further the archaea community as they are potential candidates as chemolithoautotrophs in a such a system as the subterranean ecosystem in Iceland.

### Amphipod microbiome

Bacteria associated with *Crangonyx islandicus* differed from the microbes obtained from glass beads and the spring water. These bacteria may reflect potential food items and/or symbiotic OTUs, such as normal flora, parasites and/or epibionts. Two genera of Gammaproteobacteria, *Halomonas* of the family Halomonadaceae and *Shewanella* of the family Shewanellaceae were restricted to and the far most abundant OTUs in the amphipod samples (90.7%).

The genus *Halomonas* includes 21 species which are chemoorganotrophic and have been isolated from saline environments around the world [71]. Here we observed 99% similarity to two taxa of the genus *Halomonas*: *Halomonas phoceae* (AY922995.1) in rodent blood [72] and in jellyfish rich areas in the ocean [73], and an uncultured *Halomonas* (KC337225.1) found in the intestines of roach (*Rutilus rutilus*). A more detailed assignment requires longer sequences to be assessed. Other groups of *Halomonas*, similar to our sequences (98%), are *Halomonas salifodinae* (KJ808582.1), isolated from basaltic rock and *Halomonas profundus* (NR_114956.1, 97%) found in the carapace of the deep-sea hydrothermal vent shrimp *Ramicaris exoculate* [74].

The *Shewanella*, a chemolithoheterotrophic bacteria, can utilize sulphate and reduce iron and manganese, even without being in direct contact with the metal [75]. Most species can grow at 4°C [76], which is the same as the temperature of the spring waters where the amphipods are found in. *Shewanella* has been isolated from a wide variety of samples, including freshwater and freshwater sediments [76]. Some species are known for being chitinolytic [77], indicating that they could utilize the exoskeleton of the amphipod as an energy source. This group of bacteria has been found in microbial communities growing on non-hydrothermal basaltic sea floor [78] and *Shewanella basaltis* was isolated from basalt sand [79]. The *Shewanella* might be a link in the food web between the amphipods and the basalt lava which dominates Icelandic bedrocks, utilizing minerals that are easily weathered out of this soluble bedrock type [80]. In all studied amphipod samples, two distinct types of *Shewanella* were found (Table 5) although the DNA fragment was too short for species identification. The two sequence types showed 99% similarity to five species within the genus and 98% similarity to each other. The presence of two related species could suggest symbiosis of the bacteria and the amphipods. In the groundwater amphipod genus *Niphargus* in the Frassasi cave system in Italy two phylotypes of the sulphur-oxidizing *Thiothrix* bacteria have been found. One grows as biofilm in the sulphur rich water and the second as symbionts found on hairs and spines on the appendixes of the amphipods [23, 24]. Symbiosis of chemoautotrophic bacteria and metazoans has evolved several times in extreme environments, both marine [81–84] and freshwater [20]. Further work is needed to evaluate whether the relationships between the different types of *Shewanella* and the amphipods present an example of such symbiotic evolution.

A few bacteria taxa were shared by the amphipods and the glass beads, these group may form a biofilm given either biotic or artificial support. Bacteria, which amphipods may feed on, could be expected to show up in the environmental samples (water samples and glass bead samples) whereas the symbiotic taxa should be expected to be unique for the amphipod samples or at least in low density in the environment.

Although the groundwater ecosystems in Iceland might harbour chemoautotrophy, it is also likely that the inhabitants are utilizing allochthonous material derived from the surface and historically the view of groundwater and cave ecosystem has been that organic matter from external sources are the main drivers behind the food web [16]. Icelandic lava fields are porous, and nutrients may enter groundwater system from the soil and vegetation with precipitation. However, chemical analyses have deemed this not to be in high quantities [43] and such external nutrient input does not explain primary production and food supply supporting food web including a macroinvertebrate such as the groundwater amphipod *C*. *islandicus* during the glacial periods of the Ice ages.

## Conclusion

Using environmental DNA metabarcoding targeting bacterial diversity in cold springs in lava fields within the volcanic active zone in Iceland we found that dominating taxa in terms of relative abundance are *Flavobacterium*, *Alkanindiges* and *Pseudomonas*. The bacterial richness in this habitat is shaped by pH level while the differences among springs are influenced by temperature, presence of fish and the geographical areas. This indicates that the communities in springs are both shaped by environmental factors and dispersal limitations where diversification or stochastic drift may play a role. We also demonstrate a distinct community of bacteria living with the amphipod *C*. *islandicus*, from spring water. Further studies are needed to reveal if they are a part of chemolithoautotrophic food chain that may sustain their survival even during the cold periods of Ice Age in subglacial refugia.

## Supporting information

S1 Table Chemical measurements from the spring sources. Chemical measurements from the springs. 3a&b: Þingvallavatn, 4: Miðhúsaskógur, 6: Galtalækur, 7: Kirkjubæjarklaustur, 9: Svartárvatn, 11: Sandur and 13: Klapparós. Numbers of springs refer to map locations (Fig 1).
(DOCX)

S2 Table Cell count summary. Mean number of cells ml^-1^ in both sample types with standard deviation (sd) and number of samples (n).
(DOCX)

S3 Table Bacteria taxa in all samples. List of bacteria taxa obtained in this study with mean sequence number for each sample type with standard deviation. Taxa in bold are more abundant than 20% of all samples.
(DOCX)

S4 Table Permanova results excluding warm springs. Source of variance for microbial communities in cold springs when warm water springs (number 2.W.15 and 8.W.15) were excluded.
(DOCX)
