# Supplementary material for: Bacterial diversity in Icelandic cold spring sources and in relation to the groundwater amphipod Crangonyx islandicus
Source: PLoS One. 2019 Oct 2;14(10):e0222527. doi: 10.1371/journal.pone.0222527 (PMC6774475; doi:10.1371/journal.pone.0222527)
Supplement: S1 Table — Chemical measurements from the springs. 3a&b: Þingvallavatn, 4: Miðhúsaskógur, 6: Galtalækur, 7: Kirkjubæjarklaustur, 9: Svartárvatn, 11: Sandur and 13: Klapparós. Numbers of springs refer to map locations (Fig 1). (DOCX) [file pone.0222527.s001.docx]

**Table S1 Chemical measurements from the spring sources**. 3a&b: Þingvallavatn, 4: Miðhúsaskógur, 6: Galtalækur, 7: Kirkjubæjarklaustur, 9: Svartárvatn, 11: Sandur and 13: Klapparós. Numbers of springs refer to map locations (Fig 1).

| Chemicals | Unit | 3a | 3a | 3b | 3b | 4 | 6 | 7 | 9 | 11 | 13 |
| --- | --- | --- | --- | --- | --- | --- | --- | --- | --- | --- | --- |
| B | ug/l | 2.80 | 1.35 | 3.34 | 3.39 | 1.56 | 18.22 | 6.14 | 5.17 | 8.41 | 4.10 |
| C | mg/l | 5.76 | 4.74 | 5.34 | 5.51 | 5.83 | 6.39 | 7.48 | 8.41 | 8.61 | 6.65 |
| N | mg/l | 3425.44 | 3519.88 | 3496.83 | 3542.99 | 3439.11 | 3429.44 | 3498.58 | 3550.72 | 3563.50 | 3498.07 |
| Na | mg/l | 5.13 | 1.56 | 5.75 | 5.34 | 5.34 | 17.97 | 8.01 | 10.07 | 15.67 | 8.27 |
| Mg | mg/l | 0.92 | 0.47 | 1.12 | 1.13 | 0.56 | 3.19 | 4.08 | 2.35 | 4.29 | 1.59 |
| Al | ug/l | 42.51 | 46.30 | 28.22 | 23.17 | 67.96 | 11.73 | 16.75 | 20.60 | 6.93 | 10.26 |
| Si | mg/l | 1.16 | 1.81 | 1.66 | 1.34 | 1.45 | 2.64 | 2.17 | 1.09 | 1.27 | 1.36 |
| P | ug/l | 30.97 | 25.70 | 26.39 | 33.93 | 27.33 | 89.81 | 53.79 | 60.45 | 76.55 | 40.28 |
| S | mg/l | 1.82 | 1.56 | 1.88 | 1.84 | 1.81 | 4.09 | 6.35 | 3.47 | 3.94 | 2.08 |
| Cl | mg/l | 2.66 | 0.91 | 3.65 | 3.27 | 2.07 | 4.74 | 2.86 | 1.01 | 8.24 | 5.35 |
| K | ug/l | 331.04 | 108.57 | 374.06 | 353.97 | 232.34 | 912.89 | 411.69 | 505.16 | 995.91 | 450.90 |
| Ca | mg/l | 2.38 | 1.95 | 2.11 | 2.08 | 1.98 | 5.65 | 7.33 | 3.73 | 5.25 | 4.45 |
| Sc | ng/l | 9.70 | 12.94 | 11.32 | 19.01 | 15.77 | 21.02 | 22.23 | 16.17 | 15.36 | 12.13 |
| V | ug/l | 20.32 | 8.08 | 19.23 | 19.13 | 26.52 | 22.52 | 13.93 | 59.38 | 13.11 | 13.22 |
| Cr | ng/l | 1200.00 | 603.31 | 974.59 | 709.09 | 1530.00 | 358.74 | 173.44 | 544.50 | 323.15 | 361.16 |
| Mn | ng/l | 536.77 | 239.63 | 156.19 | 139.65 | 73.86 | 151.78 | 424.59 | 197.73 | 484.53 | 106.21 |
| Fe | ug/l | 3.10 | 1.94 | 10.19 | 6.86 | 2.07 | 1.95 | 12.95 | 2.29 | 4.95 | 2.04 |
| Co | ng/l | 6.09 | 2.21 | 2.77 | 1.50 | 2.06 | 4.27 | 9.10 | 4.03 | 12.58 | 5.38 |
| Ni | ng/l | 164.72 | 47.73 | 20.17 | 30.25 | 42.01 | 96.13 | 184.55 | 97.48 | 285.77 | 64.53 |
| Cu | ng/l | 249.40 | 108.48 | 131.89 | 163.37 | 106.81 | 355.71 | 928.20 | 511.33 | 4700.00 | 437.60 |
| Zn | ug/l | 5.57 | 2.66 | 0.24 | 0.31 | 0.95 | 2.18 | 2.54 | 1.44 | 66.33 | 0.97 |
| Ga | ng/l | 766.76 | 852.99 | 590.47 | 502.98 | 1350.00 | 186.08 | 189.54 | 257.06 | 96.49 | 167.90 |
| Ge | ng/l | 40.09 | 26.46 | 40.09 | 32.07 | 56.92 | 24.05 | 19.24 | 41.69 | 32.87 | 44.10 |
| As | ng/l | 79.37 | 27.56 | 93.71 | 67.25 | 57.33 | 427.79 | 117.96 | 127.88 | 263.49 | 61.74 |
| Se | ug/l | 1.38 | 0.90 | 1.34 | 1.53 | 1.42 | 1.74 | 1.78 | 2.01 | 1.55 | 0.98 |
| Br | ug/l | 27.33 | 9.06 | 30.68 | 29.22 | 21.11 | 45.59 | 22.57 | 14.81 | 80.19 | 52.62 |
| Rb | ug/l | 1.70 | 0.50 | 1.78 | 1.63 | 1.14 | 2.49 | 2.57 | 1.55 | 2.03 | 1.44 |
| Sr | ug/l | 7.84 | 6.05 | 8.40 | 8.34 | 6.46 | 43.32 | 31.93 | 13.43 | 25.89 | 13.13 |
| Zr | ng/l | 2.77 | 2.20 | 0.92 | 2.31 | 2.66 | 5.09 | 11.10 | 3.93 | 7.74 | 3.35 |
| Mo | ng/l | 88.39 | 19.85 | 71.72 | 72.09 | 68.16 | 1180.00 | 316.79 | 339.86 | 444.86 | 85.20 |
| Ag | ng/l | 1.24 | 0.94 | 0.89 | 1.06 | 1.18 | 1.48 | 0.71 | 1.24 | 1.48 | 1.30 |
| Sn | ng/l | 3420.00 | 4300.00 | 4.93 | 6.30 | 1680.00 | 2400.00 | 1440.00 | 1280.00 | 635.29 | 558.16 |
| Sb | ng/l | 11.57 | 7.15 | 7.15 | 5.89 | 5.26 | 18.93 | 20.41 | 14.73 | 9.05 | 9.05 |
| I | ug/l | 5.08 | 2.82 | 4.23 | 3.83 | 4.51 | 6.32 | 3.55 | 3.51 | 5.66 | 4.62 |
| Cs | ng/l | 3.38 | 2.06 | 3.23 | 3.08 | 2.06 | 3.38 | 20.85 | 2.79 | 3.82 | 2.20 |
| Ba | ng/l | 197.30 | 187.13 | 310.55 | 302.41 | 183.74 | 271.21 | 431.27 | 197.98 | 305.12 | 251.55 |
| La | ng/l | 0.50 | 0.27 | 0.50 | 0.23 | 0.27 | 0.45 | 3.29 | 0.45 | 1.94 | 0.23 |
| Ce | ng/l | 0.73 | 0.87 | 0.45 | 0.42 | 0.87 | 0.56 | 5.04 | 0.52 | 1.95 | 0.49 |
| W | ng/l | 10.20 | 3.14 | 10.57 | 10.30 | 7.02 | 109.83 | 13.53 | 48.68 | 31.12 | 17.78 |
| Au | ng/l | 4.72 | 2.54 | 5.17 | 4.44 | 9.61 | 6.34 | 4.27 | 2.18 | 5.33 | 5.19 |
| Hg | ng/l | 1.09 | 1.09 | 1.49 | 0.89 | 0.59 | 1.19 | 0.99 | 1.39 | 0.69 | 0.69 |
| Pb | ng/l | 13.67 | 12.74 | 2.42 | 1.71 | 3.95 | 9.54 | 7.33 | 7.30 | 22.17 | 5.44 |
| U | ng/l | 1.25 | 0.80 | 1.53 | 1.30 | 0.90 | 21.06 | 10.61 | 5.57 | 6.22 | 2.53 |
| NO_2_ | mg/l | 0.01 | 0.01 | 0.01 | 0.01 | 0.01 | 0.01 | 0.01 | 0.01 | 0.01 | 0.01 |
| NO_3_^-^ | mg/l | 0.18 | 0.18 | 0.18 | 0.18 | 0.23 | 0.23 | 0.21 | 0.09 | 0.43 | 0.15 |
| PO_4_^3-^ | mg/l | 0.09 | 0.09 | 0.09 | 0.09 | 0.08 | 0.27 | 0.17 | 0.18 | 0.26 | 0.10 |
