# Supplementary material for: Bacterial diversity in Icelandic cold spring sources and in relation to the groundwater amphipod Crangonyx islandicus
Source: PLoS One. 2019 Oct 2;14(10):e0222527. doi: 10.1371/journal.pone.0222527 (PMC6774475; doi:10.1371/journal.pone.0222527)
Supplement: S2 Table — Mean number of cells ml-1 in both sample types with standard deviation (sd) and number of samples (n). (DOCX) [file pone.0222527.s002.docx]

**S2 Table. Cell count summary.** Mean number of cells ml^-1^ in both sample types with standard deviation (sd) and number of samples (n). Cell count for each location can be found at <https://doi.org/10.6084/m9.figshare.9773366.v1>.

|  | Mean | Sd | n |
| --- | --- | --- | --- |
| Glass beads | 1.9 * 10^7^ ml^-1^ | 3.3 * 10^7^ | 12 |
| Water | 5.0 * 10^6^ ml^-1^ | 9.1 * 10^6^ | 8 |
