# Supplementary material for: Bacterial diversity in Icelandic cold spring sources and in relation to the groundwater amphipod Crangonyx islandicus
Source: PLoS One. 2019 Oct 2;14(10):e0222527. doi: 10.1371/journal.pone.0222527 (PMC6774475; doi:10.1371/journal.pone.0222527)
Supplement: S3 Table — List of bacteria taxa obtained in this study with mean sequence number for each sample type with standard deviation. Taxa in bold are more abundant than 20% of all samples. (DOCX) [file pone.0222527.s003.docx]

**S3 Table. Bacteria taxa in all samples.** Bacteria taxa amplified in this study with mean sequence number for each sample type with standard deviation. Taxa in bold are more abundant than 20% of all samples. OTU-table with raw data can be found at <https://doi.org/10.6084/m9.figshare.9773366.v1>.

|  | Amphipods | |  | Water | |  | Glass beads | |
| --- | --- | --- | --- | --- | --- | --- | --- | --- |
|  | Mean | sd |  | Mean | sd |  | Mean | sd |
| Acetobacteraceae_unc | 0 | 0 |  | 0.46 | 1.66 |  | 0 | 0 |
| Achromobacter | 0 | 0 |  | 0 | 0 |  | 0.27 | 0.9 |
| Acidimicrobiales_OM1clade | 0 | 0 |  | 0.38 | 1.39 |  | 0 | 0 |
| Acidovorax | 0 | 0 |  | 0.15 | 0.55 |  | 0.45 | 1.21 |
| Acinetobacter | 7.5 | 12.6 |  | 11.62 | 41.58 |  | 0 | 0 |
| Actinomyces | 0.17 | 0.41 |  | 0 | 0 |  | 0 | 0 |
| Aeromonas | 0 | 0 |  | 3.92 | 14.14 |  | 0 | 0 |
| Aetherobacter | 0 | 0 |  | 0.54 | 1.94 |  | 0 | 0 |
| Albidiferax | 0 | 0 |  | 0.92 | 2.25 |  | 10.18 | 24.31 |
| Alcaligenaceae_unc | 0 | 0 |  | 0.62 | 2.22 |  | 0 | 0 |
| Alcanivorax | 0 | 0 |  | 0.23 | 0.83 |  | 0 | 0 |
| **Alkanindiges** | 21.5 | 29.82 |  | 7.31 | 26.35 |  | 475.09 | 612 |
| Alphaproteobacteria_SAR11clade_Surface1 | 0 | 0 |  | 24.69 | 89.03 |  | 0 | 0 |
| Alphaproteobacteria_SAR11clade_Surface2 | 0 | 0 |  | 0.15 | 0.55 |  | 0 | 0 |
| Alphaproteobacteria_SAR11clade_Surface4 | 0 | 0 |  | 0.08 | 0.28 |  | 0 | 0 |
| Anabaena | 0 | 0 |  | 1.15 | 4.16 |  | 0 | 0 |
| Aquabacterium | 1.17 | 2.86 |  | 0.08 | 0.28 |  | 87.73 | 172.56 |
| Aquaspirillum_arcticum_gr | 10.83 | 26.54 |  | 0 | 0 |  | 0 | 0 |
| Arcicella | 0 | 0 |  | 18.38 | 59.09 |  | 0.09 | 0.3 |
| Arenimonas | 0 | 0 |  | 4.08 | 13.82 |  | 0 | 0 |
| Azoarcus | 0 | 0 |  | 0 | 0 |  | 0.09 | 0.3 |
| **Bacillus** | 0.83 | 2.04 |  | 109.77 | 268.45 |  | 0 | 0 |
| Bacteriovorax | 0 | 0 |  | 0 | 0 |  | 0.45 | 1.51 |
| Balneatrix | 0 | 0 |  | 0.38 | 1.39 |  | 0 | 0 |
| Bergeyella | 2.17 | 5.31 |  | 0 | 0 |  | 0 | 0 |
| Betaproteobacteria_TRA3_20 | 0 | 0 |  | 0 | 0 |  | 0 | 0 |
| Blastomonas | 0 | 0 |  | 0.08 | 0.28 |  | 0 | 0 |
| Brevundimonas | 0.67 | 1.21 |  | 15.62 | 42.51 |  | 0 | 0 |
| Caedibacter | 4.5 | 6.25 |  | 0 | 0 |  | 0 | 0 |
| Caenarcaniphilales | 0 | 0 |  | 0.08 | 0.28 |  | 0 | 0 |
| Caldimonas | 0 | 0 |  | 0.23 | 0.83 |  | 0 | 0 |
| Candidatus_Acetothermus | 0 | 0 |  | 0.23 | 0.83 |  | 0 | 0 |
| Candidatus_Amoebophilus | 0 | 0 |  | 0 | 0 |  | 1.55 | 5.13 |
| Candidatus_Aquiluna | 0 | 0 |  | 1.08 | 3.88 |  | 0 | 0 |
| Candidatus_Azambacteria | 0 | 0 |  | 9 | 29.79 |  | 0 | 0 |
| Candidatus_Berkelbacteria | 0 | 0 |  | 0.08 | 0.28 |  | 0 | 0 |
| Candidatus_Falkowbacteria | 0 | 0 |  | 0.15 | 0.55 |  | 0 | 0 |
| Candidatus_Giovannonibacteria | 0 | 0 |  | 0.15 | 0.55 |  | 0 | 0 |
| Candidatus_Hepatincola | 1.5 | 3.67 |  | 0 | 0 |  | 0 | 0 |
| Candidatus_Hepatoplasma | 0.17 | 0.41 |  | 0 | 0 |  | 0 | 0 |
| Candidatus_Jorgensenbacteria | 0 | 0 |  | 1.62 | 4.25 |  | 0 | 0 |
| Candidatus_Magasanikbacteria | 0 | 0 |  | 0.08 | 0.28 |  | 0 | 0 |
| Candidatus_Neoehrlichia | 0 | 0 |  | 0 | 0 |  | 2.18 | 7.24 |
| Candidatus_Nomurabacteria | 0 | 0 |  | 5.62 | 15.27 |  | 0.82 | 2.71 |
| Candidatus_Omnitrophus | 0 | 0 |  | 0.08 | 0.28 |  | 0 | 0 |
| Candidatus_Paraholospora | 1.33 | 3.27 |  | 0 | 0 |  | 0 | 0 |
| Candidatus_Pelagibacter | 0 | 0 |  | 0 | 0 |  | 0 | 0 |
| Candidatus_Peribacteria | 0 | 0 |  | 1.38 | 4.99 |  | 0 | 0 |
| Candidatus_Symbiobacter | 0 | 0 |  | 0 | 0 |  | 28.55 | 82.2 |
| Candidatus_Uhrbacteria | 0 | 0 |  | 0.15 | 0.55 |  | 0 | 0 |
| Candidatus_Yanofskybacteria | 0 | 0 |  | 0 | 0 |  | 0 | 0 |
| Caulobacter | 0 | 0 |  | 0 | 0 |  | 0.82 | 1.83 |
| Cellvibrio | 0 | 0 |  | 0 | 0 |  | 31.73 | 72.59 |
| Cereibacter | 0 | 0 |  | 0 | 0 |  | 0.18 | 0.6 |
| **Chamaesiphon** | 0 | 0 |  | 51.46 | 137.34 |  | 0.27 | 0.65 |
| Chitinivibrionia_possible_order07 | 0 | 0 |  | 0.31 | 1.11 |  | 0 | 0 |
| Chlamydiales_cvE6 | 0 | 0 |  | 0 | 0 |  | 0 | 0 |
| Chlorobiales_OPB56 | 0 | 0 |  | 0 | 0 |  | 0 | 0 |
| Chloroflexi_Gitt_GS_136 | 0.17 | 0.41 |  | 0 | 0 |  | 0 | 0 |
| Chloroflexi_JG30_KF_CM66 | 0 | 0 |  | 0.77 | 2.77 |  | 0 | 0 |
| Chloroflexi_SAR202_clade | 0 | 0 |  | 0 | 0 |  | 0 | 0 |
| Chryseobacterium | 0.17 | 0.41 |  | 0 | 0 |  | 0 | 0 |
| **Citrobacter** | 0 | 0 |  | 29.54 | 96.64 |  | 0 | 0 |
| Cloacibacterium | 12 | 14.76 |  | 0 | 0 |  | 0 | 0 |
| Comamonadaceae | 0.17 | 0.41 |  | 0 | 0 |  | 0 | 0 |
| **Comamonadaceae_unc** | 1.33 | 2.07 |  | 7.77 | 19.99 |  | 45.45 | 75.97 |
| Comamonas | 6.67 | 6.06 |  | 0 | 0 |  | 0 | 0 |
| Corynebacterium | 5.67 | 13.88 |  | 0 | 0 |  | 0 | 0 |
| Corynebacterium1 | 2.83 | 6.94 |  | 0 | 0 |  | 0 | 0 |
| Cryomorphaceae_unc | 0 | 0 |  | 0 | 0 |  | 0 | 0 |
| Curvibacter | 0 | 0 |  | 0 | 0 |  | 0 | 0 |
| Cyanobacteria_SubsecIII_FamilyI | 0 | 0 |  | 0 | 0 |  | 0.91 | 1.7 |
| Cyanobacteria_SubsecIII_FamilyI_unc | 0 | 0 |  | 3.77 | 7.41 |  | 0 | 0 |
| Cyclobacteriaceae_unc | 0 | 0 |  | 1 | 3.61 |  | 0 | 0 |
| **Cytophaga** | 0 | 0 |  | 0 | 0 |  | 243.18 | 432.03 |
| **Cytophagaceae_unc** | 0 | 0 |  | 1.15 | 4.16 |  | 38.36 | 92.99 |
| Deefgea | 0.17 | 0.41 |  | 1.46 | 3.62 |  | 11.82 | 37.57 |
| Dehalococcoidia_GIF9 | 0 | 0 |  | 1.08 | 3.88 |  | 0 | 0 |
| Deinococcus | 0 | 0 |  | 26.92 | 90.86 |  | 0 | 0 |
| Delftia | 1.67 | 4.08 |  | 0 | 0 |  | 0 | 0 |
| Dependentiae | 0 | 0 |  | 0.69 | 1.8 |  | 0 | 0 |
| Desulfurellaceae_H16 | 0 | 0 |  | 0.46 | 1.66 |  | 0 | 0 |
| Devosia | 1.67 | 4.08 |  | 0 | 0 |  | 0 | 0 |
| Dietzia | 8.5 | 18.04 |  | 0 | 0 |  | 0 | 0 |
| Duganella | 0.83 | 1.33 |  | 0 | 0 |  | 0 | 0 |
| Elusimicrobia_LineageIV | 0 | 0 |  | 12.62 | 45.49 |  | 0 | 0 |
| Emcibacter | 0 | 0 |  | 0 | 0 |  | 0 | 0 |
| Enhydrobacter | 0 | 0 |  | 11.46 | 41.33 |  | 0 | 0 |
| Enterobacter | 0.5 | 1.22 |  | 0 | 0 |  | 0 | 0 |
| Erysipelotrichaceae_unc | 0 | 0 |  | 0 | 0 |  | 25.82 | 85.63 |
| Erythrobacter | 0 | 0 |  | 3.46 | 10.81 |  | 0 | 0 |
| Flammeovirgaceae_unc | 0 | 0 |  | 3.69 | 13.31 |  | 24.18 | 76.61 |
| Flavobacteriaceae_NS3a_mar_gr | 0 | 0 |  | 0 | 0 |  | 0 | 0 |
| Flavobacteriaceae_NS4_mar_gr | 0 | 0 |  | 0.08 | 0.28 |  | 0 | 0 |
| Flavobacteriaceae_NS5_mar_gr | 0 | 0 |  | 0 | 0 |  | 0 | 0 |
| **Flavobacterium** | 7 | 10.51 |  | 378.38 | 664.2 |  | 137.91 | 164.84 |
| Flexibacter | 0 | 0 |  | 0.15 | 0.55 |  | 0 | 0 |
| Fluviicola | 0 | 0 |  | 0.54 | 1.94 |  | 4.55 | 12.55 |
| Formosa | 0 | 0 |  | 0.08 | 0.28 |  | 0 | 0 |
| Gammaproteobacteria_KI89Aclade | 0 | 0 |  | 0.08 | 0.28 |  | 0 | 0 |
| Gracilibacteria | 0 | 0 |  | 7.85 | 27.99 |  | 8.18 | 25.21 |
| Granulicatella | 0 | 0 |  | 0.31 | 1.11 |  | 0 | 0 |
| Halanaerobiales_ODP1230B8_23 | 0 | 0 |  | 0 | 0 |  | 0 | 0 |
| Haliangium | 0 | 0 |  | 0 | 0 |  | 1.36 | 3.04 |
| Haliea | 0 | 0 |  | 0 | 0 |  | 0 | 0 |
| Halieaceae_OM60_NOR5clade | 0 | 0 |  | 0 | 0 |  | 0 | 0 |
| **Halomonadaceae** | 395.83 | 595.53 |  | 7.38 | 26.63 |  | 0 | 0 |
| **Halomonas** | 812.83 | 612.71 |  | 90 | 210.01 |  | 0 | 0 |
| Holosporaceae_unc | 0 | 0 |  | 0 | 0 |  | 0 | 0 |
| Hydrogenophaga | 1.33 | 3.27 |  | 0 | 0 |  | 0 | 0 |
| Hydrogenophilaceae_unc | 0 | 0 |  | 0.08 | 0.28 |  | 0 | 0 |
| Hymenobacter | 0 | 0 |  | 0 | 0 |  | 0.36 | 1.21 |
| Hyphomonadaceae_unc | 0 | 0 |  | 0.31 | 1.11 |  | 0 | 0 |
| Ilumatobacter | 0 | 0 |  | 1.46 | 4.01 |  | 0 | 0 |
| Jeotgalicoccus | 0 | 0 |  | 16.46 | 59.35 |  | 0 | 0 |
| Kocuria | 5.17 | 12.66 |  | 0 | 0 |  | 0 | 0 |
| Lactobacillus | 2.5 | 6.12 |  | 0 | 0 |  | 0 | 0 |
| Leadbetterella | 4 | 9.8 |  | 0 | 0 |  | 0 | 0 |
| Legionella | 0 | 0 |  | 0 | 0 |  | 0.09 | 0.3 |
| Leptolyngbya | 0 | 0 |  | 7.38 | 26.33 |  | 0 | 0 |
| Leptospira | 0 | 0 |  | 0 | 0 |  | 0.09 | 0.3 |
| Leptospirillum | 0 | 0 |  | 1.15 | 2.44 |  | 0.09 | 0.3 |
| Leptothrix | 0 | 0 |  | 2.85 | 10.26 |  | 0.27 | 0.9 |
| Limnohabitans | 0.5 | 0.84 |  | 1.08 | 3.88 |  | 0 | 0 |
| **Limnothrix** | 0 | 0 |  | 101.15 | 364.72 |  | 0 | 0 |
| Luteimonas | 0.17 | 0.41 |  | 0.15 | 0.55 |  | 0 | 0 |
| **Luteolibacter** | 2.33 | 3.83 |  | 47.62 | 162.47 |  | 0.55 | 1.29 |
| Lysinimonas | 0.83 | 2.04 |  | 0 | 0 |  | 0 | 0 |
| Lysobacter | 0 | 0 |  | 1.08 | 2.56 |  | 0 | 0 |
| Massilia | 0.5 | 1.22 |  | 0 | 0 |  | 0 | 0 |
| Methylobacterium | 0 | 0 |  | 13.54 | 26.37 |  | 0 | 0 |
| Methyloversatilis | 0 | 0 |  | 0.15 | 0.55 |  | 0 | 0 |
| Micrococcus | 4.17 | 4.4 |  | 0 | 0 |  | 0 | 0 |
| Microcoleus | 0 | 0 |  | 0 | 0 |  | 6.18 | 20.17 |
| Moraxellaceae_Agitococcus_lubricus_gr | 0 | 0 |  | 0 | 0 |  | 3 | 5.71 |
| Myxococcales_Blfdi19 | 0 | 0 |  | 0 | 0 |  | 0.27 | 0.65 |
| Nitrobacter | 0.17 | 0.41 |  | 0 | 0 |  | 0 | 0 |
| Nitrosomonadaceae_unc | 0 | 0 |  | 2.77 | 9.98 |  | 0 | 0 |
| Nitrospira | 0 | 0 |  | 0.08 | 0.28 |  | 0 | 0 |
| Novosphingobium | 0 | 0 |  | 2.31 | 8.02 |  | 0 | 0 |
| Oceanospirillales_SAR86_clade | 0 | 0 |  | 0 | 0 |  | 0 | 0 |
| Oceanospirillales_ZD0405 | 0 | 0 |  | 0 | 0 |  | 0 | 0 |
| Octadecabacter | 0 | 0 |  | 0 | 0 |  | 0 | 0 |
| Oligoflexales_0319_6G20 | 0.17 | 0.41 |  | 0 | 0 |  | 0 | 0 |
| **Omnitrophica** | 0 | 0 |  | 55.54 | 130.94 |  | 0 | 0 |
| Opitutae_vadinHA64 | 0 | 0 |  | 0 | 0 |  | 9.18 | 23.4 |
| Opitutus | 0 | 0 |  | 0.15 | 0.55 |  | 0 | 0 |
| Oxalobacteraceae_unc | 0 | 0 |  | 1.69 | 6.1 |  | 0.09 | 0.3 |
| Parcubacteria | 0 | 0 |  | 11 | 17.92 |  | 0 | 0 |
| Paucibacter | 0 | 0 |  | 0 | 0 |  | 10.09 | 30.3 |
| Pedobacter | 0.33 | 0.82 |  | 0.23 | 0.83 |  | 4.36 | 13.51 |
| Pelomonas | 2.67 | 4.84 |  | 0 | 0 |  | 0 | 0 |
| Peredibacter | 0 | 0 |  | 0 | 0 |  | 0 | 0 |
| Peregrinibacteria | 0 | 0 |  | 0.77 | 2.77 |  | 0 | 0 |
| **Perlucidibaca** | 1.83 | 4.49 |  | 0.08 | 0.28 |  | 68.45 | 155.36 |
| Phenylobacterium | 0 | 0 |  | 0 | 0 |  | 0.27 | 0.9 |
| Phormidium | 0 | 0 |  | 5.77 | 20.5 |  | 0 | 0 |
| Planktomarina | 0 | 0 |  | 0.23 | 0.83 |  | 0 | 0 |
| Pleurocapsa | 0 | 0 |  | 0.08 | 0.28 |  | 0 | 0 |
| Polaribacter1 | 0 | 0 |  | 0 | 0 |  | 0 | 0 |
| Polaromonas | 0 | 0 |  | 19.15 | 37.65 |  | 28.91 | 33.12 |
| Polymorphobacter | 0 | 0 |  | 15.85 | 52.79 |  | 0.73 | 2.41 |
| Porticoccaceae_SAR92clade | 0 | 0 |  | 0 | 0 |  | 0 | 0 |
| Propionibacterium | 20.33 | 13.37 |  | 3 | 8.03 |  | 0 | 0 |
| Proteobacteria_ARKICE90 | 0 | 0 |  | 0 | 0 |  | 0 | 0 |
| Pseudoclavibacter | 1 | 2.45 |  | 0 | 0 |  | 0 | 0 |
| Pseudohongiella | 0 | 0 |  | 0 | 0 |  | 0.73 | 2.41 |
| **Pseudomonas** | 0.33 | 0.82 |  | 277.08 | 459.44 |  | 5.91 | 12.04 |
| Psychrobacter | 0.33 | 0.82 |  | 0 | 0 |  | 0 | 0 |
| Pusillimonas | 0 | 0 |  | 0 | 0 |  | 0 | 0 |
| Rheinheimera | 0 | 0 |  | 0 | 0 |  | 0 | 0 |
| Rhizobacter | 0 | 0 |  | 1.69 | 4.31 |  | 2 | 6.31 |
| **Rhizorhapis** | 0 | 0 |  | 1.31 | 3.5 |  | 39 | 104.32 |
| Rhodobacter | 0 | 0 |  | 2.08 | 5.17 |  | 0 | 0 |
| Rhodobacteraceae_unc | 0.83 | 2.04 |  | 1.08 | 2.5 |  | 0 | 0 |
| Rhodocyclaceae_unc | 0 | 0 |  | 3.46 | 12.48 |  | 0 | 0 |
| Rhodoferax | 0 | 0 |  | 0.31 | 0.75 |  | 106.36 | 165.18 |
| Rhodospirillaceae_AEGEAN169mar_gr | 0 | 0 |  | 0.23 | 0.83 |  | 0 | 0 |
| Rickettsiaceae_unc | 0.33 | 0.82 |  | 0 | 0 |  | 0 | 0 |
| Rickettsiales_SM2D12 | 0 | 0 |  | 1.15 | 4.16 |  | 0 | 0 |
| Roseococcus | 0 | 0 |  | 0.38 | 1.39 |  | 0 | 0 |
| Roseomonas | 0 | 0 |  | 0.08 | 0.28 |  | 0 | 0 |
| Rothia | 1.17 | 1.83 |  | 0.46 | 1.66 |  | 0 | 0 |
| Rubellimicrobium | 0 | 0 |  | 0.08 | 0.28 |  | 0 | 0 |
| Runella | 0 | 0 |  | 0 | 0 |  | 0 | 0 |
| **Sandaracinaceae_unc** | 0 | 0 |  | 0 | 0 |  | 68 | 206.42 |
| Sandaracinus | 0 | 0 |  | 0 | 0 |  | 3.27 | 7.38 |
| Saprospiraceae_unc | 0 | 0 |  | 1 | 3.61 |  | 0 | 0 |
| Schlesneria | 0 | 0 |  | 6.54 | 23.57 |  | 0 | 0 |
| Sedimentitalea | 0 | 0 |  | 0 | 0 |  | 0 | 0 |
| **Shewanella** | 466.33 | 61.19 |  | 35 | 87 |  | 0 | 0 |
| Shigella | 0 | 0 |  | 16.08 | 57.97 |  | 0.64 | 1.8 |
| Simkaniaceae_unc | 0 | 0 |  | 7.23 | 26.07 |  | 0 | 0 |
| Sphingobacteriales_envOPS17 | 0 | 0 |  | 0 | 0 |  | 1.36 | 3.23 |
| Sphingobacteriales_NS11_12mar_gr | 0 | 0 |  | 0 | 0 |  | 0.55 | 1.81 |
| Sphingobacteriales_PHOS_HE51 | 0 | 0 |  | 0 | 0 |  | 0 | 0 |
| Sphingobacteriales_unc | 0 | 0 |  | 0.77 | 2.77 |  | 0 | 0 |
| Sphingobium | 0 | 0 |  | 126.85 | 189.28 |  | 0 | 0 |
| Sphingomonadaceae_unc | 0 | 0 |  | 0.77 | 2.77 |  | 12.64 | 34.55 |
| Sphingomonadales_SWB04 | 0 | 0 |  | 0.38 | 1.39 |  | 0 | 0 |
| Sphingomonas | 0 | 0 |  | 78.46 | 169.69 |  | 0 | 0 |
| Sphingopyxis | 0 | 0 |  | 0.92 | 3.33 |  | 2.55 | 8.44 |
| Sphingorhabdus | 0 | 0 |  | 0.15 | 0.38 |  | 0 | 0 |
| SR1_Absconditabacteria | 0 | 0 |  | 4 | 14.12 |  | 0 | 0 |
| Staphylococcus | 3.5 | 6.44 |  | 1.15 | 3.87 |  | 0 | 0 |
| **Stenotrophomonas** | 0 | 0 |  | 78.54 | 120 |  | 0 | 0 |
| Streptococcus | 5.67 | 9.73 |  | 0 | 0 |  | 0 | 0 |
| Sulfitobacter | 0 | 0 |  | 4.77 | 17.2 |  | 0 | 0 |
| Sulfuritalea | 0 | 0 |  | 0 | 0 |  | 0.18 | 0.6 |
| Tabrizicola | 0 | 0 |  | 3.69 | 13.31 |  | 0 | 0 |
| Terrimicrobium | 0 | 0 |  | 0 | 0 |  | 0 | 0 |
| Thermotalea | 0 | 0 |  | 4 | 14.42 |  | 0 | 0 |
| Thermus | 0.5 | 1.22 |  | 0 | 0 |  | 0 | 0 |
| Turneriella | 0 | 0 |  | 0 | 0 |  | 0 | 0 |
| **Tychonema** | 0 | 0 |  | 3.69 | 13.31 |  | 267.64 | 563.26 |
| Undibacterium | 0 | 0 |  | 0.08 | 0.28 |  | 19.36 | 59.04 |
| Variovorax | 0 | 0 |  | 0 | 0 |  | 0.09 | 0.3 |
| Verrucomicrobia_OPB35_soil_gr | 0 | 0 |  | 0 | 0 |  | 0 | 0 |
| Verrucomicrobia_S_BQ2_57soil_gr | 0 | 0 |  | 0 | 0 |  | 0 | 0 |
| Verrucomicrobiaceae_unc | 0 | 0 |  | 1.92 | 6.93 |  | 0.91 | 2.21 |
| Vogesella | 5.67 | 9.33 |  | 0 | 0 |  | 0 | 0 |
| Wolbachia | 0 | 0 |  | 4.23 | 15.25 |  | 0.45 | 1.51 |
| WWE3 | 0 | 0 |  | 0.23 | 0.83 |  | 0 | 0 |
| Xanthomonadaceae_unc | 0 | 0 |  | 0.54 | 1.94 |  | 0.18 | 0.6 |
| Xylella | 4 | 7.04 |  | 0 | 0 |  | 0 | 0 |
