# Supplementary material for: Bacterial diversity in Icelandic cold spring sources and in relation to the groundwater amphipod Crangonyx islandicus
Source: PLoS One. 2019 Oct 2;14(10):e0222527. doi: 10.1371/journal.pone.0222527 (PMC6774475; doi:10.1371/journal.pone.0222527)
Supplement: S4 Table — Source of variance for microbial communities in cold springs when warm water springs (number 2.W.15 and 8.W.15) were excluded. (DOCX) [file pone.0222527.s004.docx]

**S4 Table. Permanova results excluding warm springs.** Source of variance for microbial communities in cold springs when warm water springs (samples 2.W.15 and 8.W.15) were excluded.

| Source of variation | df | SS | MS | F | R2 | p |
| --- | --- | --- | --- | --- | --- | --- |
| Sample type | 1 | 1.4 | 1.4 | 5.6 | 0.16 | 0.001 |
| Source/surface | 1 | 0.6 | 0.6 | 2.5 | 0.07 | 0.003 |
| Spring type | 1 | 0.4 | 0.4 | 1.7 | 0.05 | 0.057 |
| Temp | 1 | 0.6 | 0.6 | 2.3 | 0.06 | 0.008 |
| Elevation | 1 | 0.3 | 0.3 | 1.0 | 0.03 | 0.418 |
| Fish | 1 | 0.7 | 0.7 | 2.6 | 0.08 | 0.001 |
| Area | 4 | 2.1 | 0.5 | 2.1 | 0.23 | 0.001 |
| Residuals | 11 | 2.8 | 0.3 |  | 0.31 |  |
| Total | 21 | 8.8 |  |  | 1.00 |  |
